# PySimi: a unified framework for similarity measure evaluation in spectral clustering with applications to omics data

**DOI:** 10.3389/fgene.2026.1913487

**Published:** 2026-08-19

**Authors:** Tianyi Shi, Xiucai Ye, Zeng Zou, Wenyu Xi, Tetsuya Sakurai

**Affiliations:** Department of Computer Science, University of Tsukuba, Tsukuba, Japan

**Keywords:** clustering analysis, omics data analysis, similarity measure, spectral clustering, transcriptomics

## Abstract

High-throughput omics technologies generate increasingly large and complex datasets, creating a growing demand for clustering methods capable of identifying meaningful biological patterns. Spectral clustering is widely used for analyzing high-dimensional omics data, but its performance strongly depends on the construction of the similarity matrix. Although numerous similarity measures have been proposed, most existing spectral clustering tools support only a limited set of similarity construction strategies, making systematic evaluation and comparison difficult. Here, we present PySimi, an open-source Python framework for flexible similarity matrix construction and spectral clustering. PySimi integrates classical, adaptive, and neighborhood-based similarity measures within a unified and extensible framework and provides a consistent workflow for constructing, comparing, and evaluating similarity matrices. The framework also supports downstream analyses, including dimensionality reduction and visualization, and offers an interactive web application for exploratory analysis. We evaluated PySimi using multiple bulk and single-cell RNA-sequencing datasets. The results demonstrate that the choice of similarity measure can substantially influence clustering outcomes and downstream biological interpretation. While no single method consistently achieved the best performance across all datasets, adaptive and neighborhood-based approaches generally showed stronger performance than classical methods. By providing a unified platform for similarity matrix construction, comparison, and evaluation, PySimi enables systematic investigation of similarity measures and facilitates their application to diverse omics datasets.

## 1 Introduction

High-throughput transcriptomic technologies, including bulk RNA sequencing (RNA-seq) and single-cell RNA sequencing (scRNA-seq), have become indispensable tools for investigating biological heterogeneity in complex diseases and cellular systems (Rappoport and Shamir, 2018; Tzec-Interián et al., 2025). The rapid accumulation of high-dimensional omics data has created increasing demand for computational methods capable of identifying meaningful biological patterns (Han et al., 2025). Among these methods, clustering plays a central role in disease subtyping, cell-type identification, and exploratory analysis by grouping samples or cells with similar molecular characteristics (Chauvel et al., 2020; Brière et al., 2021).

Spectral clustering has emerged as a powerful approach for analyzing high-dimensional biological data because of its ability to capture non-linear and complex data structures (John et al., 2020; Rappoport and Shamir, 2019; Shi et al., 2023; Qi et al., 2021; Nagumo et al., 2025; Zhang et al., 2022; Shi et al., 2024). Unlike traditional clustering algorithms such as K-means (Bach and Jordan, 2003), spectral clustering operates on a graph representation of the data, where pairwise relationships among samples are encoded in a similarity matrix (Beauchemin, 2015). The similarity matrix defines the graph structure from which the graph Laplacian and spectral embedding are derived, making it a critical component of the clustering process (Ye and Sakurai, 2017). Consequently, the quality of spectral clustering depends not only on the clustering algorithm itself but also on how similarities between samples are defined. Different similarity measures may capture substantially different data structures, potentially leading to markedly different clustering outcomes and biological interpretations.

A wide variety of similarity measures have been proposed for spectral clustering. Classical approaches include correlation-based and kernel-based methods such as Pearson correlation (Romero et al., 2022; Fitch et al., 2019; Lodi et al., 2024), Spearman correlation (Zhang et al., 2023; Fotuhi Siahpirani et al., 2016), cosine similarity (Chen, 2018; Yin and Sun, 2022), and Gaussian kernels (Von Luxburg, 2007; Favati et al., 2020; Ye et al., 2023). More recently, adaptive similarity measures (Ye and Sakurai, 2017; Zhang et al., 2011; Ye and Sakurai, 2018) have been developed to account for local density variations and heterogeneous neighborhood structures. Neighborhood-based approaches, including shared-nearest-neighbor (SNN) methods (He et al., 2015; Ye and Sakurai, 2016; Ye and Sakurai, 2015; Zelnik-Manor and Perona, 2004), further improve local structure preservation by incorporating neighborhood information into similarity estimation. Because these approaches characterize data relationships from different perspectives, their effectiveness often depends on dataset-specific properties, including dimensionality, sparsity, noise levels, and biological heterogeneity.

Despite the growing number of similarity construction methods, their practical use remains limited. Most existing spectral clustering tools provide only a small number of predefined similarity functions, typically relying on Gaussian kernels or k-nearest-neighbor graphs. As a result, researchers often adopt default similarity settings without systematically evaluating alternative similarity definitions despite their potential impact on clustering performance. Furthermore, many adaptive and neighborhood-based similarity measures are implemented in separate software packages or research-specific code repositories, making them difficult to access, compare, and evaluate within a unified analytical workflow. This limitation is particularly important in omics studies, where the most suitable similarity measure may vary substantially across datasets and biological applications.

To address these challenges, we developed PySimi, an open-source Python framework for flexible similarity matrix construction and spectral clustering. PySimi integrates a diverse collection of classical, adaptive, and neighborhood-based similarity measures within a unified and extensible framework, enabling users to construct, compare, and evaluate similarity matrices through a consistent workflow. In addition to a Python API, PySimi provides an interactive web application that supports similarity matrix generation, spectral clustering, visualization, and exploratory analysis without requiring programming expertise.

The main contributions of this work are threefold. First, we develop a unified framework that integrates diverse similarity construction methods for spectral clustering. Second, the framework provides both programmatic and web-based interfaces for similarity construction, clustering analysis, and visualization. Third, we systematically evaluate the implemented similarity measures using multiple bulk and single-cell RNA-seq datasets, providing practical insights into the influence of similarity construction strategies on clustering performance and biological interpretation. By enabling systematic comparison of similarity measures within a unified environment, PySimi facilitates method selection and supports reproducible omics data analysis.

## 2 Package description

### 2.1 Package overview

PySimi was developed to facilitate the systematic construction, comparison, and evaluation of similarity measures for spectral clustering. The framework provides a unified environment in which users can generate similarity matrices using a diverse collection of classical, adaptive, and neighborhood-based methods and directly apply them to downstream analyses. By standardizing the workflow across different similarity construction strategies, PySimi enables consistent comparison of clustering performance under different similarity definitions.

**FIGURE 1:**
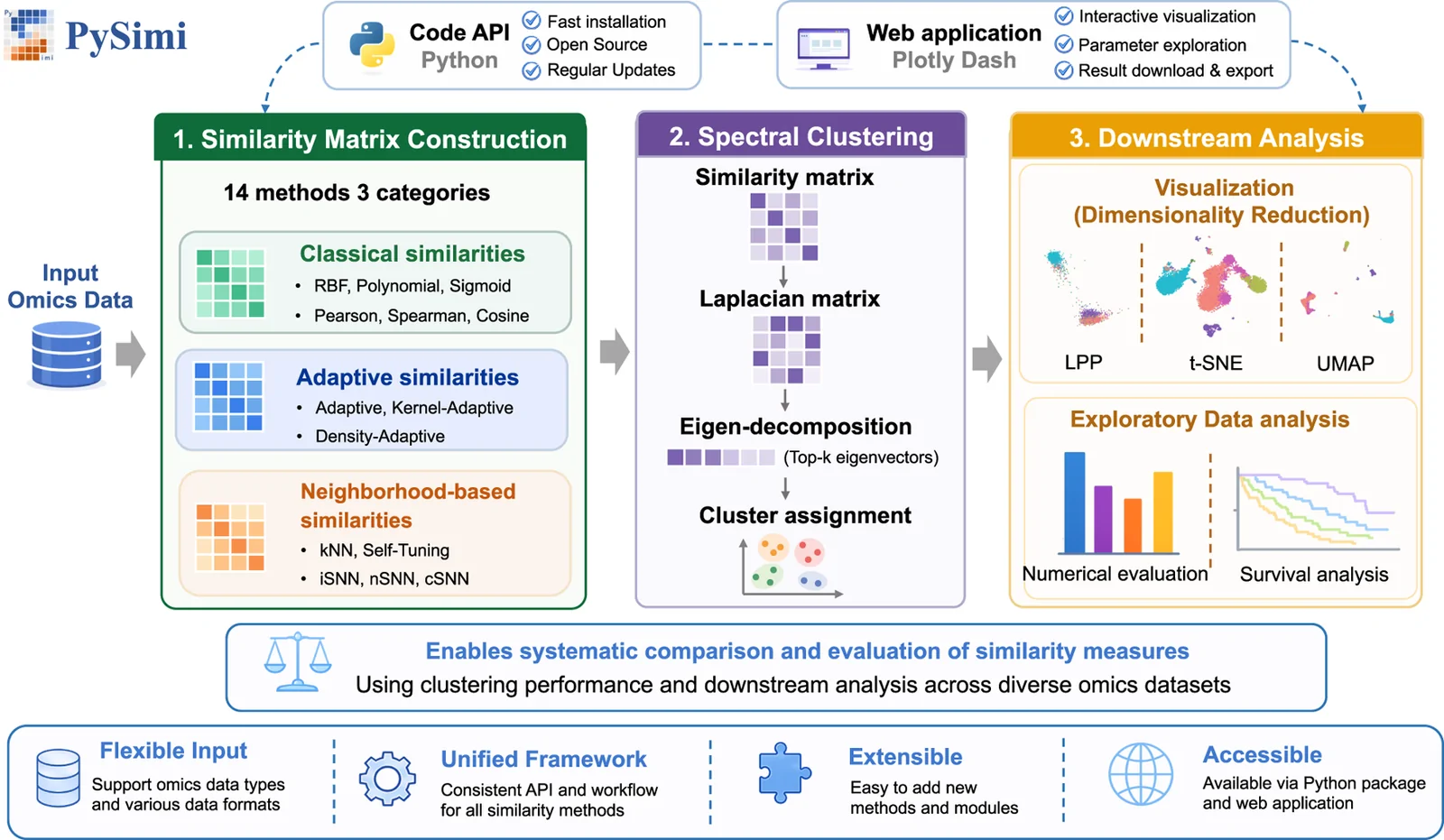
Overview of the PySimi workflow for similarity-based omics data analysis. PySimi constructs similarity matrices using classical, adaptive, and neighborhood-based methods. The matrices produced are primarily used for spectral clustering and can also be applied to embedding-based analysis for visualization and exploratory data analysis.

As illustrated in Figure 1, PySimi integrates similarity matrix construction, spectral clustering, dimensionality reduction, and visualization within a single framework. The generated similarity matrices can be used for spectral clustering and similarity-preserving embedding methods, including locality preserving projection (LPP) (He and Niyogi, 2003), t-SNE (Van der Maaten and Hinton, 2008), and UMAP (McInnes et al., 2018). PySimi supports both programmatic and graphical interfaces, allowing users to perform analyses through Python scripts or an interactive web application.

PySimi provides a unified workflow for similarity matrix construction, spectral clustering, and downstream analysis, enabling systematic comparison of similarity measures within a consistent analytical framework.

### 2.2 Supported similarity measures

PySimi currently implements 14 similarity construction methods spanning three major categories (Table 1): classical, adaptive, and neighborhood-based approaches. These methods were selected to represent diverse strategies for characterizing sample relationships and to facilitate systematic evaluation of similarity construction techniques in spectral clustering applications. Table 1 summarizes the implemented similarity measures and their corresponding categories.

**TABLE 1:** Summary of the similarity construction methods implemented in PySimi.

Category	Methods
Classical	Pearson, Spearman, cosine, RBF, polynomial, and sigmoid
Adaptive	Adaptive (Ye and Sakurai, 2017), kernel-adaptive (Ye and Sakurai, 2018), and density-adaptive (Zhang et al., 2011)
Neighborhood-based	kNN (Von Luxburg, 2007), self-tuning (Zelnik-Manor and Perona, 2004), iSNN (He et al., 2015), nSNN (Ye and Sakurai, 2016), and cSNN (Ye and Sakurai, 2015)

### 2.3 Software information

PySimi is implemented in Python (v3.9.6) and follows a modular and extensible software architecture. The framework is built on widely used scientific computing libraries, including NumPy (v2.0.2), SciPy (v1.13.1), scikit-learn (v1.6.1), pandas (v2.3.3), and UMAP (v0.5.11). Similarity construction methods, spectral clustering algorithms, and downstream analytical modules are organized into independent components, facilitating future extension and maintenance.

All functions in PySimi follow a consistent API design, enabling reproducible analyses across different datasets and parameter settings. The package is open-source and freely available through GitHub (https://github.com/Tianyi-Shi-Tsukuba/PySimi) and the Python Package Index (PyPI) (https://pypi.org/project/pysimi-toolkit), where installation instructions and usage examples are provided.

### 2.4 Web application

To improve accessibility for users without programming experience, PySimi provides an interactive web application for similarity matrix construction and exploratory analysis. The application allows users to upload datasets, generate similarity matrices using different methods, perform spectral clustering, and visualize clustering results through an intuitive graphical interface. The web interface is implemented using Dash (Hossain et al., 2019) and runs directly in any modern web browser without additional configuration. It is available at https://d7a8ef74-a727-46b4-8a9a-b7735a08bad0.plotly.app.

**FIGURE 2:**
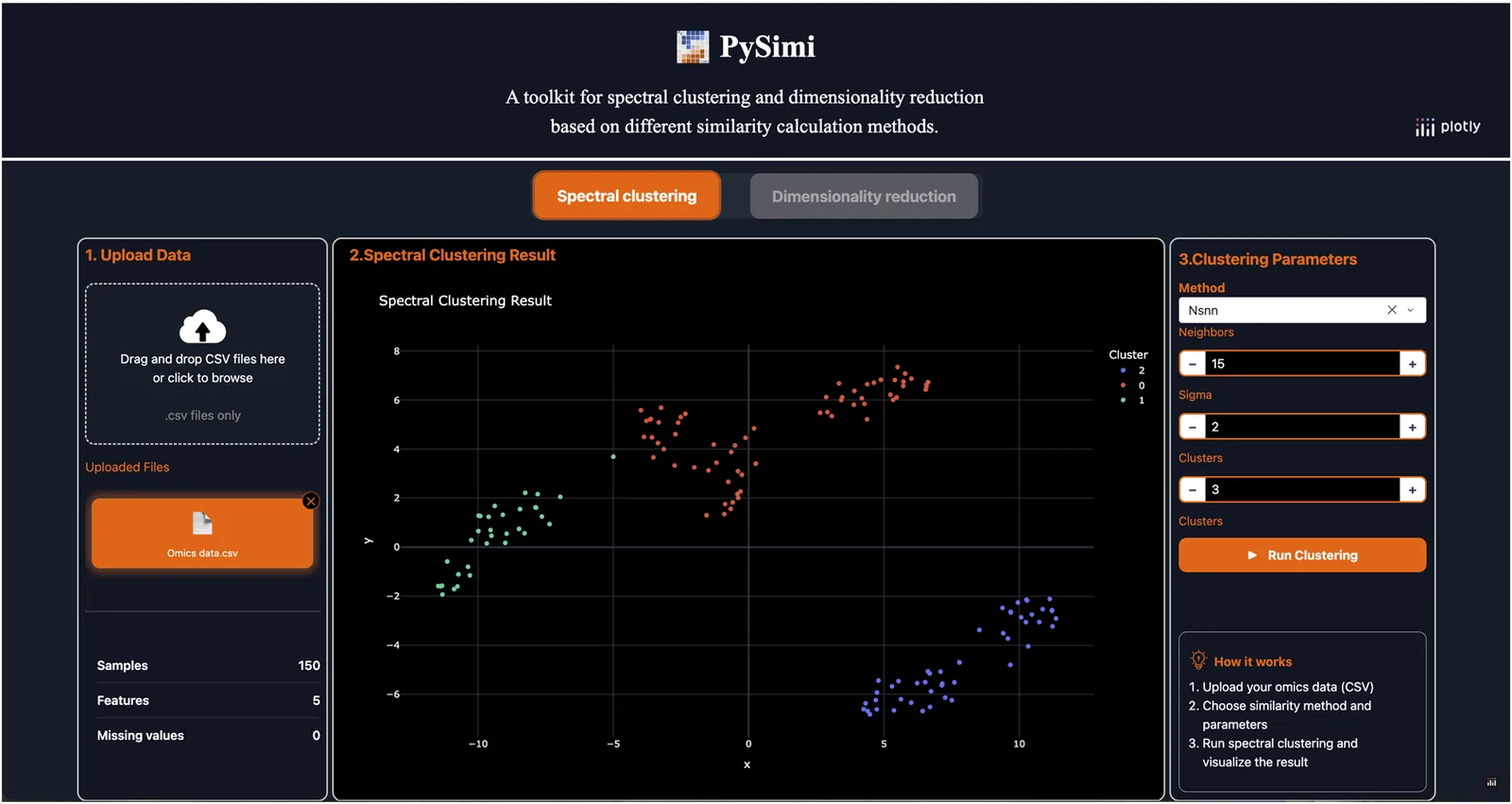
Example of the PySimi web application interface for interactive similarity matrix construction and visualization.

The web application also supports dimensionality reduction and interactive visualization, enabling users to explore data structures and compare the effects of different similarity measures in real-time. By complementing the Python package with a graphical interface, PySimi facilitates rapid method evaluation, educational demonstration, and exploratory omics data analysis. An example of the web application interface is shown in Figure 2.

## 3 Methodology

The core functionality of PySimi is the construction of similarity matrices for spectral clustering. To facilitate systematic evaluation of similarity measures, PySimi implements a diverse collection of similarity construction methods spanning three major categories: classical, adaptive, and neighborhood-based approaches (Table 1). Given an input data matrix $X={\left[x_{1},x_{2},\ldots ,x_{n}\right]}^{T}\in R^{n\times d}$ , where n and d denote the number of samples and features, respectively. All methods construct a symmetric similarity matrix $S\in R^{n\times n}$ , where each entry $s_{ij}$ quantifies the similarity between samples $x_{i}$ and $x_{j}$ . The resulting similarity matrices can be directly used for spectral clustering and downstream analyses. The corresponding similarity measures are defined in Equations 1–27.

### 3.1 Similarity matrix construction

#### 3.1.1 Classical similarities

Classical similarity measures estimate pairwise relationships directly from distances, correlations, or kernel functions. These methods are computationally efficient and are widely used as baseline approaches in spectral clustering.

1. Pearson correlation similarity

Pearson similarity captures linear relationships between samples and is widely used in gene expression and co-expression analyses. Each feature vector is standardized by

$${\tilde{x}}_{i}=\frac{x_{i}-\mu_{i}}{\sigma_{i}},$$ (1)

where $\mu_{i}$ and $\sigma_{i}$ denote the mean and standard deviation of $x_{i}$ , respectively. The Pearson similarity is then defined as:

$$s_{ij}^{P\text{earson}}=\frac{1}{d}{\tilde{x}}_{i}^{T}{\tilde{x}}_{j}.$$ (2)

2. Spearman correlation similarity

Spearman similarity is based on rank correlation and is generally more robust to non-normal distributions and outliers than Pearson correlation. Each feature vector is first transformed into ordinal ranks:

$$r_{i}=\text{rank}\left(x_{i}\right),$$ (3)

and then standardized as

$${\tilde{r}}_{i}=\frac{r_{i}-{\bar{r}}_{i}}{\text{std}\left(r_{i}\right)}.$$ (4)

The Spearman similarity is defined as

$$s_{ij}^{\text{Spearman}}=\frac{1}{d}{\tilde{r}}_{i}^{T}{\tilde{r}}_{j}.$$ (5)

3. Gaussian (RBF) similarity

RBF similarity transforms Euclidean distances into affinities through exponential distance decay and is one of the most commonly used similarity functions in spectral clustering. Let $D_{ij}=∥x_{i}-x_{j}{∥}_{2}$ denote the pairwise Euclidean distance. The Gaussian similarity is defined as

$$s_{\text{ij}}^{\text{RBF}}=\exp\left(‐\frac{{D_{\text{ij}}}^{2}}{2\sigma^{2}}\right),$$ (6)

where σ is a bandwidth parameter.

4. Cosine similarity

Cosine similarity measures the angular similarity between feature vectors and is particularly useful for high-dimensional data; it is defined as

$$s_{ij}^{\text{Cosine}}=\frac{x_{i}^{T}x_{j}}{∥x_{i}{∥}_{2}∥x_{j}{∥}_{2}},$$ (7)

where $∥\cdot {∥}_{2}$ denotes the $l_{2}$ norm.

5. Polynomial kernel similarity

Polynomial kernel similarity captures non-linear relationships through higher-order feature interactions. It is defined as

$$s_{ij}^{\text{Poly}}={\left(\gamma x_{i}^{T}x_{j}+c_{0}\right)}^{p},$$ (8)

where p is the polynomial degree, γ is a scaling parameter, and $c_{0}$ is a bias term. By default, γ=1/d .

6. Sigmoid kernel similarity

Sigmoid kernel provides non-linear similarity estimation inspired by neural network activation functions, which is defined as

$$s_{ij}^{\text{Sigmoid}}=\tanh\left(\gamma x_{i}^{T}x_{j}+c_{0}\right).$$ (9)

#### 3.1.2 Adaptive similarities

Adaptive similarity measures adjust the affinity estimation according to local data characteristics, such as neighborhood structure or density variation. These methods aim to improve similarity estimation in heterogeneous datasets where global similarity definitions may be insufficient.

1. Adaptive neighbor similarity

For each data point $x_{i}$ , a probability vector $p_{i}$ is obtained by solving

$$\min_{p_{i}}\sum_{j=1}^{n}p_{ij}{D_{ij}}^{2},s.t.\;\sum_{j=1}^{n}p_{ij}=1,0\leq p_{ij}\leq 1.$$ (10)

The closed-form solution is given by

$$p_{ij}=\frac{D_{i,\left(k+1\right)}^{2}-{D_{ij}}^{2}}{kD_{i,\left(k+1\right)}^{2}-\sum_{m=1}^{k}D_{i,m}^{2}},$$ (11)

where only the k -nearest neighbors have non-zero probabilities. The symmetric similarity matrix is defined as

$$s_{ij}^{\text{Adaptive}}=\frac{p_{ij}+p_{ji}}{2}.$$ (12)

This method learns adaptive neighborhood weights directly from local sample relationships.

2. Kernel-adaptive neighbor similarity

Kernel-adaptive similarity extends adaptive neighborhood estimation to kernel space, enabling more flexible modeling of non-linear data structures. In the kernel space, the pairwise distance is defined as

$$d_{ij}^{\left(K\right)}=K_{ii}+K_{jj}-2K_{ij},$$ (13)

where $K_{ij}$ is the Gaussian (RBF) kernel that is calculated as defined in Equation 6. The adaptive weights are defined as

$$s_{ij}=\frac{d_{i,\left(k+1\right)}-d_{ij}^{\left(K\right)}}{kd_{i,\left(k+1\right)}-\sum_{m=1}^{k}d_{i,\left(m\right)}},\text{for }x_{j}\in N_{k}\left(i\right),$$ (14)

and $s_{ij}=0$ otherwise. Here, $N_{k}\left(i\right)$ denotes the set of k -nearest neighbors of $x_{i}$ in the kernel space. The symmetric kernel-adaptive similarity is defined as

$$s_{ij}^{\text{KernelAdaptive}}=\frac{s_{ij}+s_{ji}}{2}.$$ (15)

3. Local density adaptive similarity

This method incorporates local density information to improve affinity estimation in datasets with uneven sample distributions. Let $N_{i}$ denote the ε -neighborhood of $x_{i}$ , where the ε -neighborhood of a point represents the hypersphere centered at that point with radius ε . The common-near-neighbor (CNN) measure of $x_{i}$ and $x_{j}$ is defined as

$$C_{ij}=\left|N_{i}\cap N_{j}\right|.$$ (16)

The local density adaptive similarity is then defined as

$$S_{ij}^{\text{DensityAdaptive}}=\exp\left(-\frac{D_{ij}^{2}}{2\sigma^{2}\left(C_{ij}+1\right)}\right).$$ (17)

#### 3.1.3 Neighborhood-based similarities

Neighborhood-based similarity measures characterize relationships through local graph structures rather than direct pairwise distances. By incorporating neighborhood information, these methods are often effective for high-dimensional and noisy datasets.

1. k-nearest neighbor (kNN) similarity

kNN similarity constructs sparse affinity graphs by preserving local neighborhood connections. The k NN similarity is defined as

$$s_{ij}=\left\{\begin{array}{ll} \;1, & x_{j}\in N_{i},\\ \;0, & \text{otherwise}. \end{array}\right.$$ (18)

The similarity matrix is constructed as

$$s_{ij}^{\text{kNN}}=\frac{1}{2}\left(s_{ij}+s_{ji}\right).$$ (19)

2. Self-tuning similarity

Self-tuning similarity utilizes sample-specific scaling parameters to adapt affinity estimation to local density variations. A local scaling parameter is defined as

$$\sigma_{i}=D_{i,\left(k\right)},$$ (20)

where $D_{i,\left(k\right)}$ denotes the distance from $x_{i}$ to its k -th nearest neighbor. The self-tuning similarity is defined as

$$s_{ij}^{\text{SelfTuning}}=\exp\left(-\frac{D_{ij}^{2}}{\sigma_{i}\sigma_{j}}\right).$$ (21)

3. Importance of shared nearest neighbors (iSNN) similarity

iSNN similarity incorporates the importance of shared neighbors to improve robustness against noisy connections. An adjacency matrix B is constructed using a global threshold, such that

$$B_{ij}=\left\{\begin{array}{ll} 1, & D_{ij}<\theta ,\\ 0, & \text{otherwise}, \end{array}\right.$$ (22)

where θ is set as the mean of all pairwise distances. Two vectors, namely, $h_{i}$ and $a_{i}$ , are iteratively updated based on B , and the importance score of each sample is defined as

$${Im}_{i}=h_{i}+a_{i},i=1,\ldots ,n.$$ (23)

Let $\sigma_{i}$ denote the distance to its k -th nearest neighbor. The iSNN similarity is then defined as

$$s_{ij}^{\text{iSNN}}=\exp\left(-\frac{D_{ij}^{2}}{\left(1+\alpha \;\max_{u\in N_{i}\cap N_{j}}{Im}_{u}\right)\;\sigma_{i}\;\sigma_{j}}\right),$$ (24)

where α>0 is a regularization parameter.

4. Number of shared nearest neighbors (nSNN) similarity

nSNN similarity quantifies relationships according to the number of shared neighbors between samples. The neighbor set $N_{i}$ is determined based on the same RBF kernel matrix. The nSNN similarity is defined as

$$s_{ij}^{\text{nSNN}}=\frac{\left|N_{i}\cap N_{j}\right|}{k}.$$ (25)

5. Closeness of shared nearest neighbors (cSNN) similarity

cSNN extends nSNN by additionally considering the relative proximity of shared neighbors. To incorporate the relative closeness of shared neighbors, a rank-based weight is assigned. Let $r_{i}\left(x\right)$ denote the rank of a shared neighbor $x\in N_{i}$ . The unnormalized similarity between $x_{i}$ and $x_{j}$ is defined as

$${\tilde{S}}_{ij}=\sum_{x\in N_{i}\cap N_{j}}\left(k-r_{i}\left(x\right)+1\right)\left(k-r_{j}\left(x\right)+1\right).$$ (26)

The normalized cSNN similarity is given by

$$S_{ij}^{\text{cSNN}}=\frac{{\tilde{S}}_{ij}}{\max\left(\tilde{S}\right)}.$$ (27)

### 3.2 Spectral clustering

Given a similarity matrix $S\in R^{n\times n}$ constructed using any of the methods described above, spectral clustering is performed using a common workflow. First, the degree matrix D is computed from the similarity matrix S , that is, $D=\text{diag}\left(d_{1},\ldots ,d_{n}\right)$ , where $d_{i}=\sum_{j=1}^{n}S_{ij}$ . The normalized graph Laplacian is then constructed as Equation 28:

$$L_{sym}=I-D^{-\frac{1}{2}}SD^{-\frac{1}{2}},$$ (28)

and eigenvalue decomposition is performed to obtain a low-dimensional spectral embedding.

The eigenvectors corresponding to the smallest eigenvalues are used to construct the embedding matrix $U=\left[u_{1},u_{2},\ldots ,u_{c}\right]\in R^{n\times c},$ where $u_{1},u_{2},\ldots ,u_{c}$ denote the c smallest eigenvectors. Finally, K-means clustering is applied to the row vectors of U to obtain cluster assignments.

Because all similarity matrices are processed using the same spectral clustering pipeline, differences in clustering performance primarily reflect the influence of similarity construction strategies rather than downstream clustering procedures. This design enables systematic evaluation and comparison of similarity measures within a unified analytical framework.

## 4 Experimental evaluation

### 4.1 Datasets

To evaluate the utility of PySimi for systematic comparison of similarity measures, experiments were conducted on both bulk RNA-seq and single-cell RNA-seq datasets.

For bulk RNA-seq analysis, three cancer transcriptomic datasets were obtained from The Cancer Genome Atlas (TCGA) (McLendon et al., 2008): LIHC (liver hepatocellular carcinoma), LUAD (lung adenocarcinoma), and KIRP (kidney renal papillary cell carcinoma). Gene expression values were log-transformed to reduce the influence of extreme expression values, and genes were ranked according to the coefficient of variation (CV). The top 50% most variable genes were retained to reduce noise while preserving sufficient biological variation for downstream analyses.

For single-cell RNA-seq analysis, three datasets were included: an in-house dataset (Wang et al., 2020), PBMC10k, and CBMC8k. No additional batch-effect correction was performed in this study. Data preprocessing followed the standard Seurat workflow (Stuart et al., 2019). Expression matrices were normalized using log-normalization to stabilize the expression distribution, and highly variable genes were identified using the variance-stabilizing transformation (VST) method.

The key characteristics of the preprocessed datasets are summarized in Table 2. These datasets represent diverse omics analysis scenarios with varying sample sizes, feature dimensions, and biological complexity, providing a suitable benchmark for evaluating different similarity construction strategies.

**TABLE 2:** Summary of the preprocessed bulk and single-cell RNA-seq datasets.

Dataset	Data type	Number of samples	Number of features
LIHC	Bulk	342	5,133
KIRP	Bulk	246	5,133
LUAD	Bulk	475	5,133
Inhouse	Single-cell	1,372	2,000
PBMC10k	Single-cell	7,865	2,000
CBMC8k	Single-cell	8,617	2,000

### 4.2 Evaluation metrics and experimental setup

All similarity construction methods implemented in PySimi were evaluated using the datasets described above. For each similarity measure, a similarity matrix was first constructed and subsequently used for spectral clustering. The evaluation metrics are defined in Equations 29–34.

#### 4.2.1 Bulk RNA-seq analysis

For bulk RNA-seq datasets, clustering performance was evaluated from both the biological and clinical perspectives. Biological relevance was assessed using survival significance based on the *p*-value from the log-rank test.

$$S_{\text{survival}}=-\log_{10}\left(p\right).$$ (29)

Larger values of $S_{\text{survival}}$ indicate stronger survival differences among clusters. A threshold of p<0.05 (i.e., $S_{\text{survival}}>1.3$ ) is considered statistically significant.

Clinical relevance was evaluated according to the number of significantly enriched clinical variables. Six variables were considered, including age, gender, and four pathological features (tumor size, lymph node status, distant metastasis, and pathological stage). Statistical significance was assessed using the Kruskal–Wallis test (McKight and Najab, 2010) for continuous variables and the chi-square test (Tallarida and Murray, 1987) for categorical variables. The clinical relevance score is defined as the number of clinical variables with statistically significant *p*-values.

$$E_{\text{clinical}}=\sum_{i=1}^{6}1\left(p_{i}<0.05\right),$$ (30)

where $1\left(\cdot \right)$ is the indicator function. Larger values of $E_{\text{clinical}}$ indicate stronger clinical relevance of the clusters.

Because the optimal number of clusters is generally unknown in cancer subtype analysis, clustering was performed using three commonly adopted settings (k = 3, 4, and 5), and the reported results correspond to the average across these settings.

#### 4.2.2 Single-cell RNA-seq analysis

For single-cell RNA-seq datasets, clustering performance was evaluated using four standard external clustering metrics, including normalized mutual information (NMI), adjusted Rand index (ARI), clustering accuracy (ACC), and purity.

NMI is defined as

$$\text{NMI}\left(y,c\right)=\frac{2I\left(y;c\right)}{H\left(y\right)+H\left(c\right)},$$ (31)

where y and c are the ground-truth and predicted labels, respectively, $I\left(\cdot ;\cdot \right)$ denotes mutual information, and $H\left(\cdot \right)$ denotes entropy.

ARI is defined as

$$\text{ARI}\left(y,c\right)=\frac{\text{RI}-E\left[\text{RI}\right]}{\max\left(\text{RI}\right)-E\left[\text{RI}\right]},$$ (32)

where RI denotes the Rand index.

ACC is defined as

$$\text{ACC}=\frac{1}{n}\sum_{i=1}^{n}1\left(y_{i}=\pi \left(c_{i}\right)\right),$$ (33)

where $y_{i}$ and $c_{i}$ are the true and predicted labels of sample i and $\pi \left(\cdot \right)$ denotes the optimal label permutation obtained via the Hungarian algorithm.

Purity measures the extent to which each predicted cluster contains samples from a single ground-truth class, and it is defined as

$$\text{Purity}=\frac{1}{n}\sum_{k}\max_{j}\left|C_{k}\cap T_{j}\right|,$$ (34)

where $C_{k}$ denotes the set of samples in the k -th predicted cluster and $T_{j}$ denotes the set of samples in the j -th true class.

The number of clusters was determined according to annotated cell types. To account for stochastic variation, each experiment was repeated 10 times using different random seeds, and the mean ± standard deviation was reported.

For visualization, clustering results were projected into low-dimensional space using locality preserving projection (LPP), t-SNE, and UMAP. Hyperparameters associated with similarity construction methods, including kernel bandwidths and neighborhood sizes, were selected according to the dataset characteristics and method requirements.

#### 4.2.3 Hyperparameter settings

Similarity construction methods implemented in PySimi mainly require two types of hyperparameters: the kernel bandwidth ( σ ) and the neighborhood size ( k ).

For methods requiring a kernel bandwidth parameter, σ was set to the median of all pairwise Euclidean distances in the dataset. This data-dependent heuristic provides an adaptive scaling factor while avoiding manual parameter tuning across different datasets.

For methods requiring a neighborhood size parameter, the neighborhood size was selected according to the characteristics of each dataset. Smaller neighborhood sizes were used for smaller datasets, while larger neighborhood sizes were adopted for larger datasets to ensure stable estimation of local neighborhood structure.

### 4.3 Clustering results

#### 4.3.1 Bulk RNA-seq results

**FIGURE 3:**
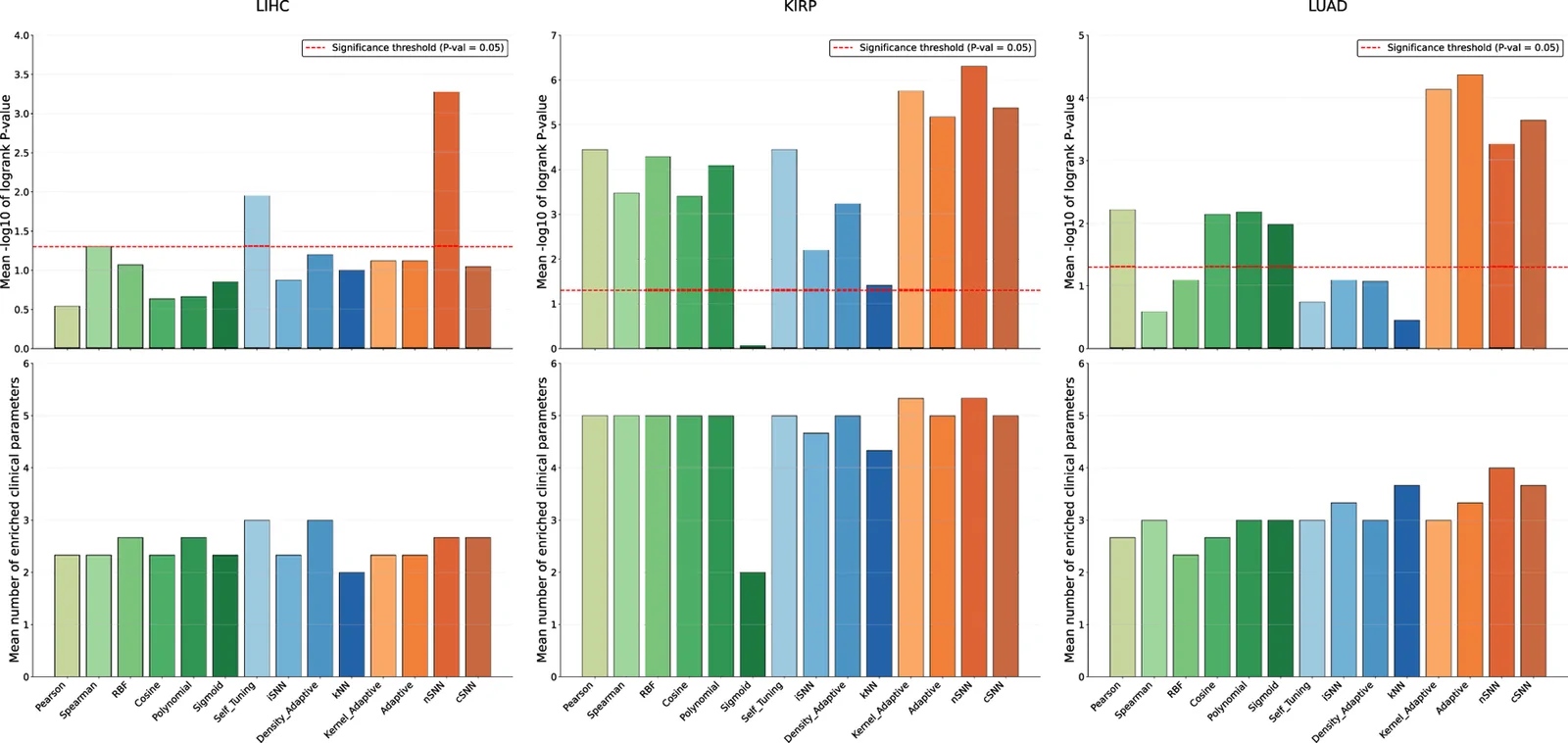
Comparison of clustering performance across different similarity methods on bulk RNA-seq datasets. The upper row shows the mean survival significance score $S_{\text{survival}}$ , averaged over 3, 4, and 5 clusters for LIHC, KIRP, and LUAD. The red dashed line indicates the significance threshold. The lower row shows the mean number of significantly enriched clinical parameters $E_{\text{clinical}}$ . Higher values indicate better performance.

Figure 3 summarizes the clustering performance of different similarity measures on the three TCGA datasets. Overall, substantial performance differences were observed among similarity measures. Adaptive and neighborhood-based approaches generally achieved stronger survival separation while maintaining comparable or improved clinical parameter enrichment relative to classical similarity measures. In the LIHC dataset, only a subset of similarity measures achieved statistically significant survival separation. Among them, Spearman, self-tuning, and nSNN produced relatively strong survival discrimination. Clinical parameter enrichment showed less variation across methods. In the KIRP dataset, most similarity measures produced significant survival differences among clusters. Kernel-adaptive, adaptive, nSNN, and cSNN achieved the highest survival significance scores, while also maintaining competitive clinical enrichment performance. In the LUAD dataset, kernel-adaptive, adaptive, nSNN, and cSNN again demonstrated superior survival separation. Several classical methods, including Pearson, cosine, polynomial, and sigmoid similarities, also achieved statistically significant results, although their performance was generally less consistent across datasets.

**FIGURE 4:**
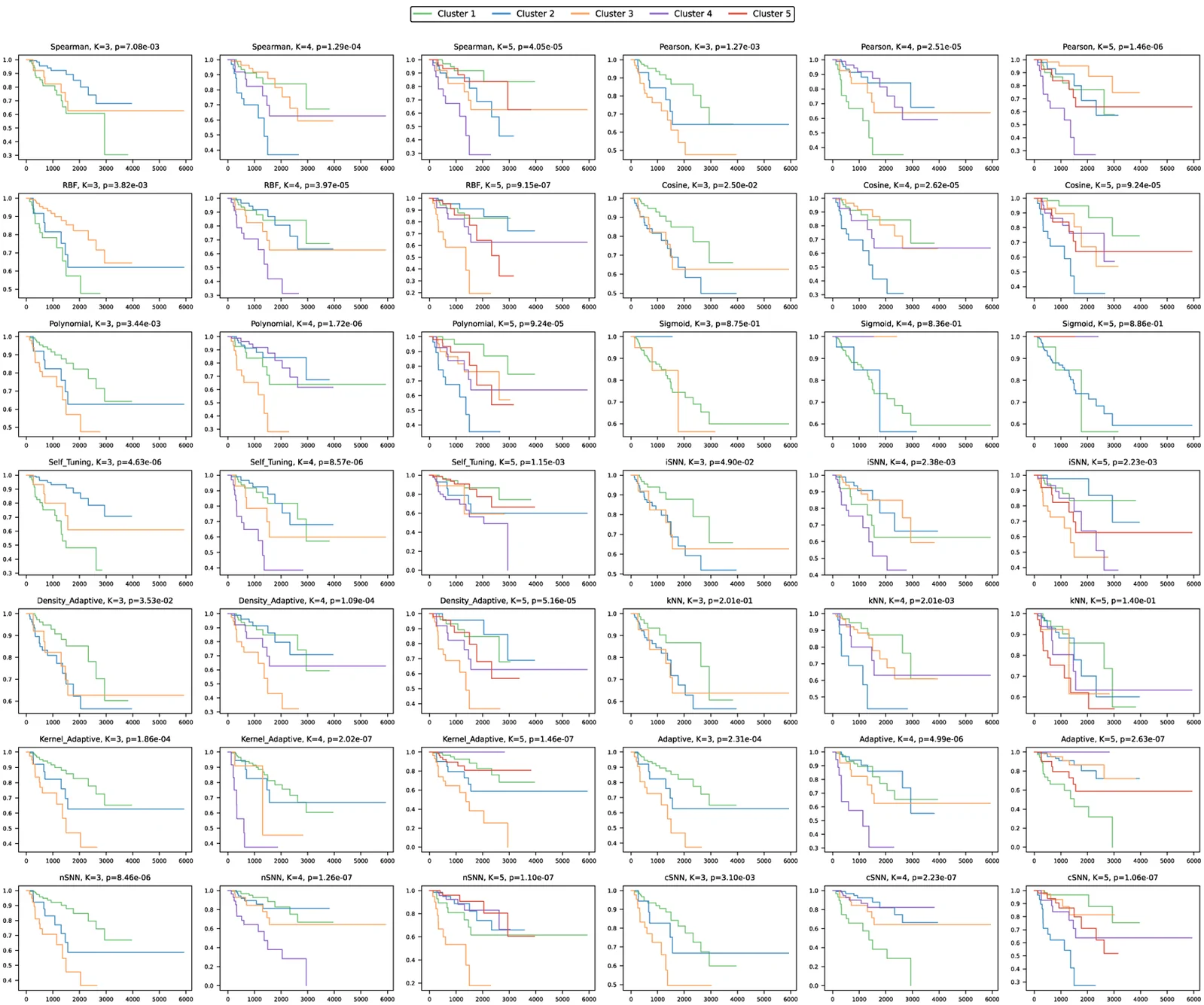
Kaplan–Meier survival curves of KIRP patients for different similarity methods and cluster numbers (k = 3, 4, and 5). Each row corresponds to a similarity method, and each column corresponds to a cluster number.

Figure 4 presents Kaplan–Meier survival curves for the KIRP dataset under different cluster settings. Although many similarity measures produced significant survival differences, the resulting subtype structures varied considerably. Neighborhood-based methods such as nSNN and cSNN showed relatively consistent survival separation across different cluster numbers, highlighting the influence of similarity construction strategies on downstream biological interpretation.

#### 4.3.2 Single-cell RNA-seq results

**FIGURE 5:**
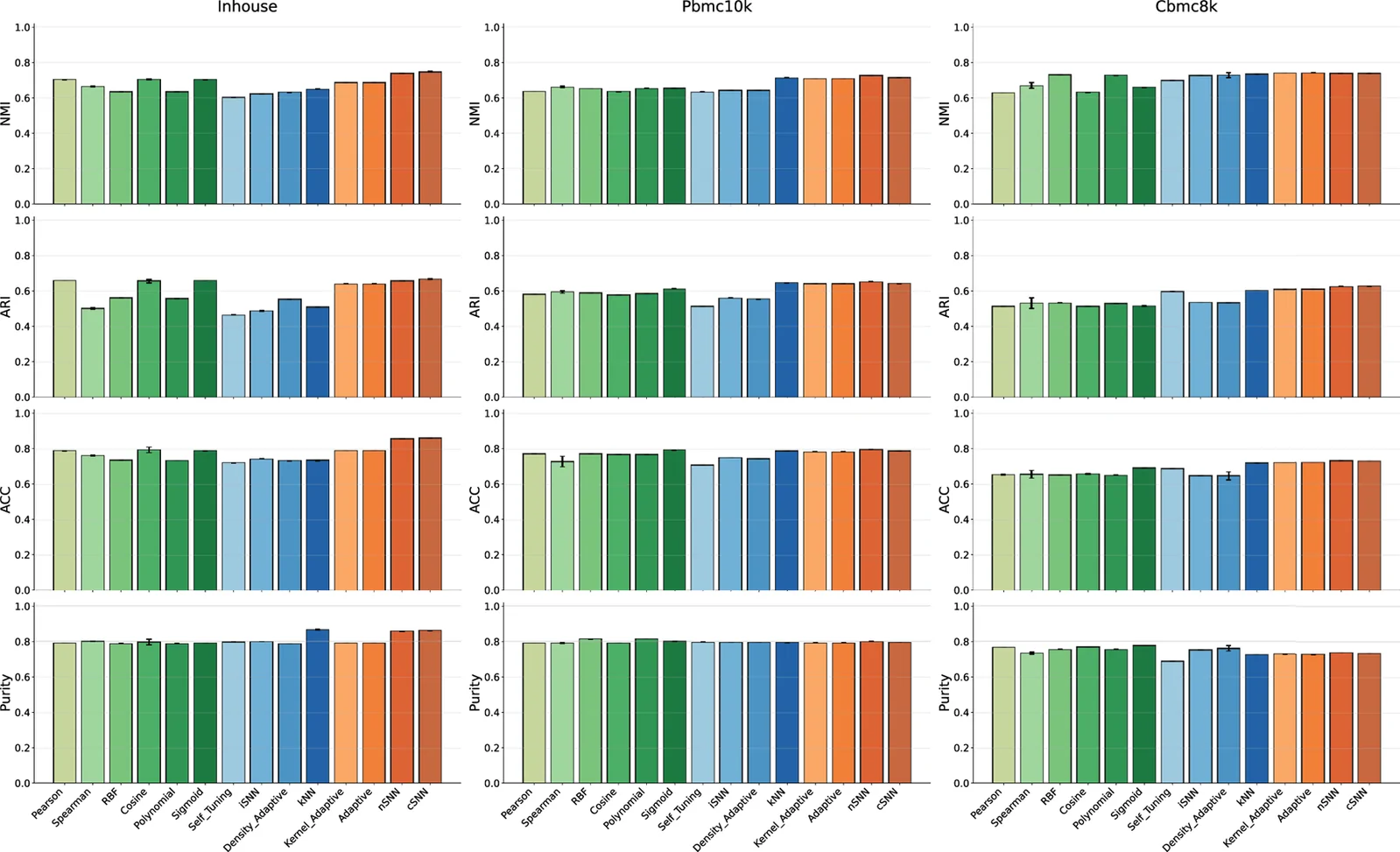
Comparison of clustering performance across different similarity methods on three single-cell RNA-seq datasets. The four rows correspond to NMI, ARI, ACC, and purity. Bars represent the mean performance across repeated runs, with error bars indicating standard deviation. Higher values indicate better agreement with annotated cell types.

Figure 5 summarizes the clustering performance across the three single-cell RNA-seq datasets using NMI, ARI, ACC, and purity. Consistent with the bulk RNA-seq analyses, substantial performance differences were observed among similarity measures. Overall, adaptive and neighborhood-based methods consistently achieved stronger agreement with annotated cell types than classical similarity measures. In particular, kernel-adaptive, adaptive, nSNN, and cSNN demonstrated competitive and stable performance across datasets and evaluation metrics. On the PBMC10k dataset, neighborhood-based approaches, including iSNN, nSNN, cSNN, and kNN, consistently outperformed most classical similarity measures. Similar trends were observed for the CBMC8k dataset, where adaptive and shared-nearest-neighbor-based methods achieved higher ACC and purity scores.

Since the neighborhood size is a key hyperparameter for similarity construction methods based on neighborhood information, we conducted a parameter sensitivity analysis to evaluate their robustness. Specifically, the neighborhood size k was varied from 10 to 50 on the in-house dataset, and the results are shown in Supplementary Figure S1. Overall, most methods exhibited relatively stable performance across different neighborhood sizes. Among them, kernel-adaptive, adaptive, nSNN, and cSNN consistently achieved competitive clustering performance. Relatively larger performance variations were observed for nSNN and cSNN across different neighborhood sizes.

To evaluate the computational cost of different similarity construction methods for large-scale single-cell RNA-seq analysis, we compared their runtime, and the results are summarized in Supplementary Table S1. Overall, classical similarity measures required the shortest computation time, while neighborhood-based and adaptive methods generally required longer computation times because of the additional neighborhood construction and local similarity estimation procedures.

**FIGURE 6:**
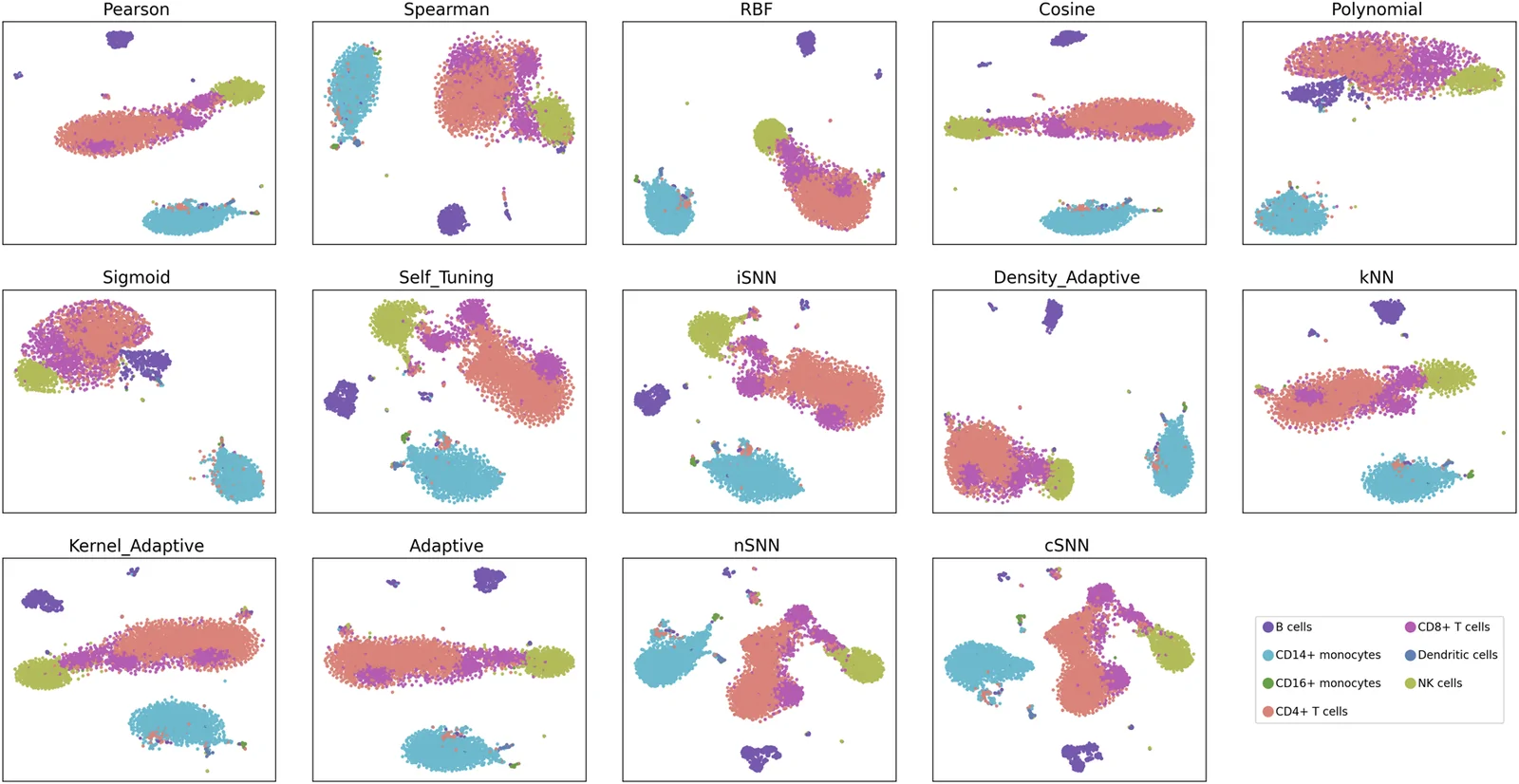
t-SNE visualizations of single-cell clustering results on the PBMC10k dataset using different similarity methods.

**FIGURE 7:**
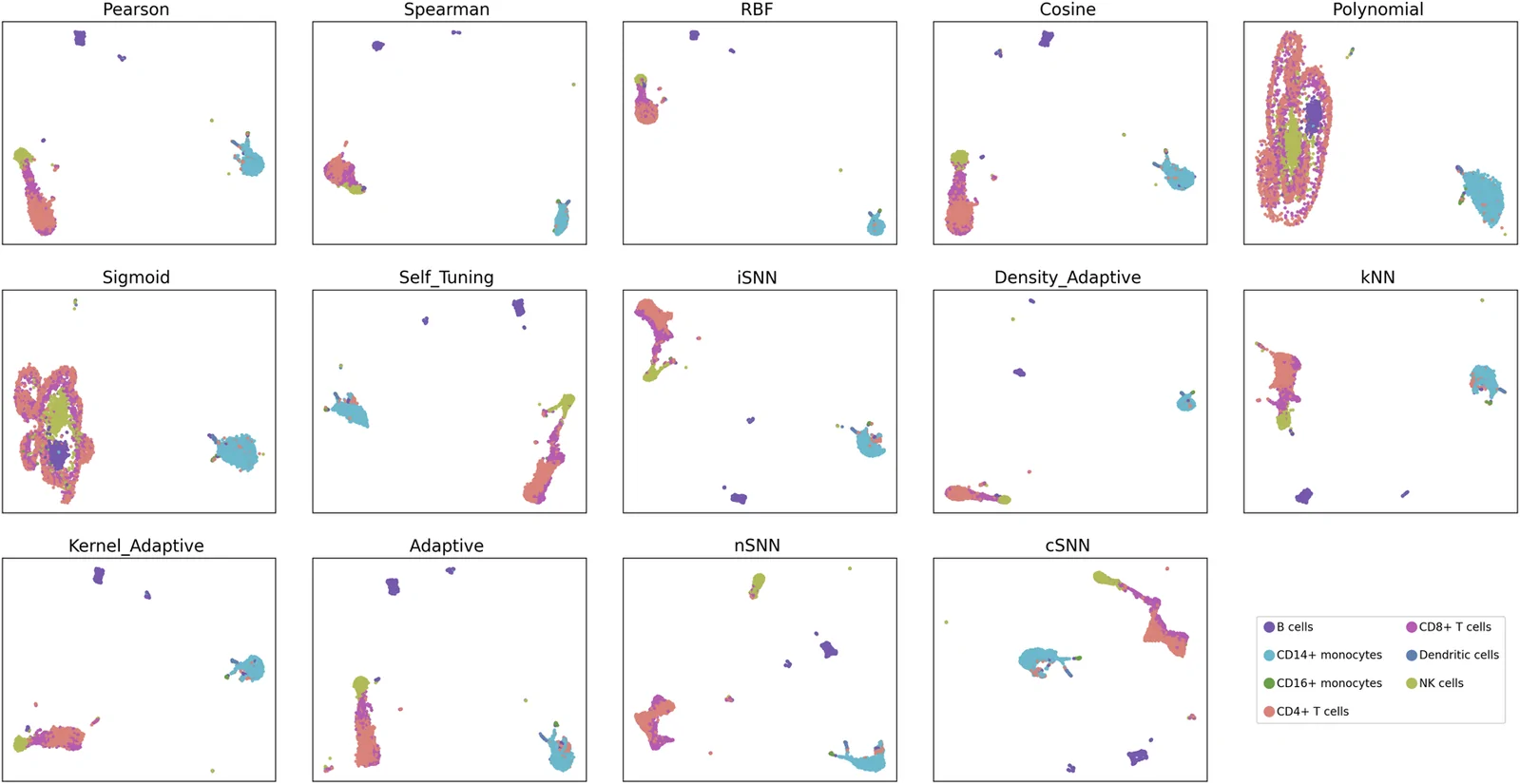
UMAP visualizations of single-cell clustering results on the PBMC10k dataset using different similarity methods.

**FIGURE 8:**
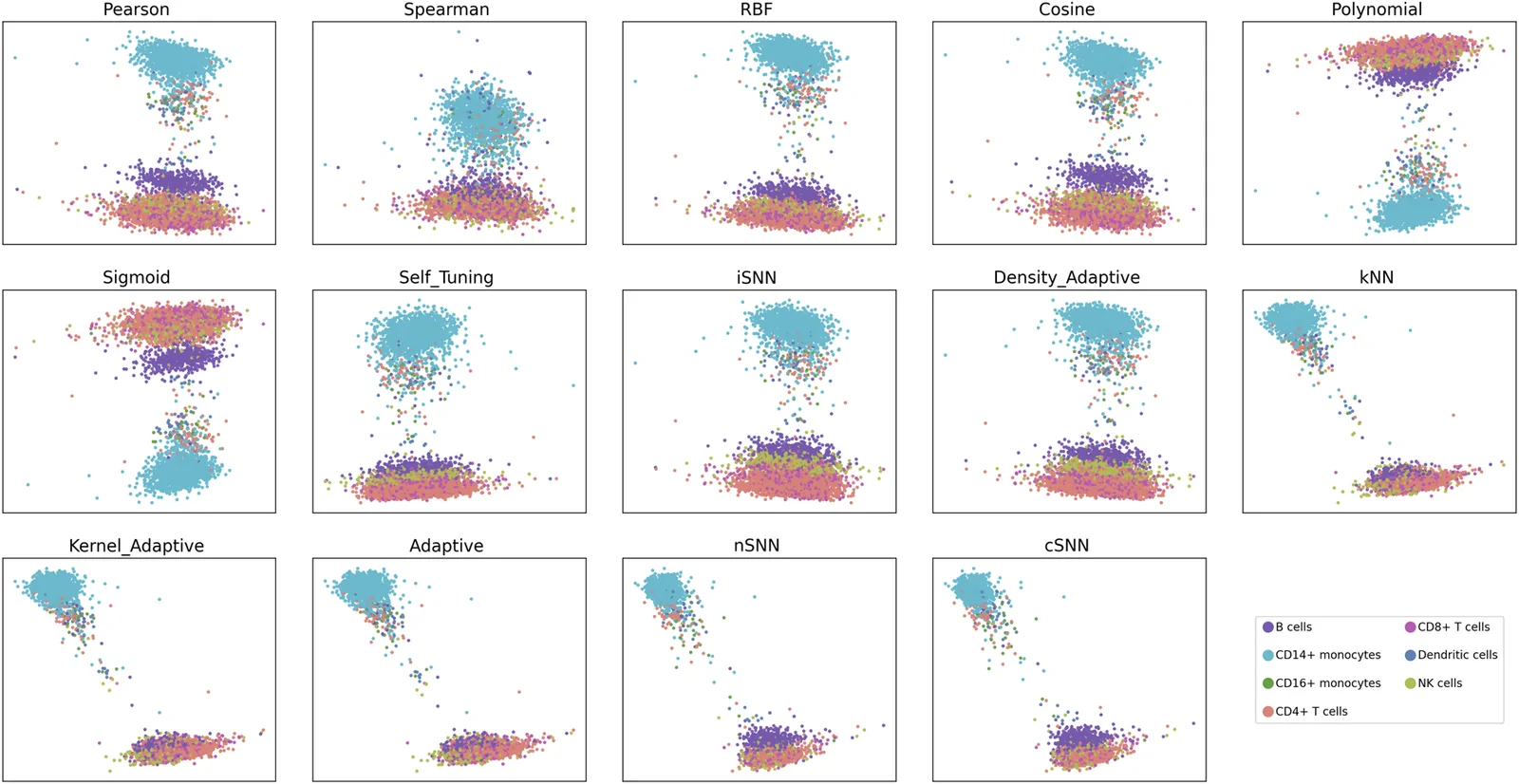
LPP visualizations of single-cell clustering results on the PBMC10k dataset using different similarity methods.

Figures 6–8 present low-dimensional visualizations of the PBMC10k dataset generated using t-SNE, UMAP, and LPP based on similarity matrices constructed by different methods. Noticeable differences in cluster compactness, separation, and structure preservation were observed among similarity measures, indicating that similarity construction can substantially influence downstream analyses beyond clustering itself.

In general, adaptive and neighborhood-based similarity measures tended to produce more coherent and well-separated cluster structures, while several classical methods led to greater overlap among closely related cell populations. Consistent with the quantitative evaluation results, kernel-adaptive, adaptive, nSNN, and cSNN frequently produced clearer cluster boundaries and better preservation of local neighborhood structures across multiple datasets and visualization techniques. Similar trends were observed in t-SNE, UMAP, and LPP embeddings, indicating that these methods are effective in capturing biologically meaningful relationships in heterogeneous omics datasets.

These findings complement the quantitative clustering results and further demonstrate the importance of similarity matrix construction in omics data analysis. By enabling direct comparison of visualization outcomes under different similarity definitions, PySimi provides a practical framework for exploratory analysis and method evaluation.

## 5 Discussion and conclusion

Similarity matrix construction is a critical component of spectral clustering and has a substantial impact on clustering performance. Although numerous similarity measures have been proposed, most existing spectral clustering tools provide only a limited set of predefined similarity functions, making systematic comparison difficult. To address this challenge, we developed PySimi, an open-source framework that integrates diverse similarity construction strategies within a unified and extensible environment for omics data analysis.

A key contribution of PySimi is its ability to facilitate systematic evaluation of similarity measures under a consistent analytical workflow. The framework integrates 14 similarity construction methods spanning classical, adaptive, and neighborhood-based categories and provides both Python and web-based interfaces for constructing similarity matrices, performing spectral clustering, and visualizing clustering results. By enabling direct comparison of different similarity measures under identical clustering settings, PySimi supports reproducible analyses and informed method selection for diverse omics datasets.

Using this framework, we systematically evaluated similarity measures across multiple bulk and single-cell RNA-seq datasets. The results demonstrated that the choice of similarity measure can substantially influence clustering outcomes and downstream biological interpretation. No single similarity measure consistently achieved the best performance across all datasets, highlighting the importance of selecting similarity construction strategies according to the dataset characteristics and analytical objectives. PySimi is not intended to identify a universally optimal similarity measure. Instead, the framework provides a practical environment for evaluating how different similarity definitions influence clustering outcomes in specific datasets and analytical scenarios. Nevertheless, adaptive and neighborhood-based approaches generally showed stronger and more consistent performance than classical methods. This difference in performance may be related to the biological characteristics of the datasets. For example, single-cell RNA-seq datasets typically exhibit substantial cellular heterogeneity and varying degrees of cell-type separability, while bulk transcriptomic datasets often contain heterogeneous disease subtypes with complex underlying structures. Adaptive similarity measures can better accommodate variations in local sample density, and neighborhood-based methods preserve local neighborhood relationships, enabling more effective identification of biologically meaningful groups. In contrast, classical similarity measures mainly rely on global pairwise relationships and may be less effective for datasets with high heterogeneity or limited cell-type separability. In particular, kernel-adaptive, adaptive, nSNN, and cSNN frequently achieved competitive results across multiple evaluation criteria, indicating that incorporating local neighborhood structure and density information can improve similarity estimation for complex and heterogeneous omics data.

The study also highlights the importance of systematic comparison when applying spectral clustering to biological datasets. Similarity measures that performed well in one dataset did not necessarily achieve comparable performance in others, emphasizing that similarity selection should be considered an integral part of clustering analysis rather than a fixed preprocessing step. By providing a unified platform for method comparison, PySimi enables researchers to explore these differences efficiently and transparently.

Several limitations should be acknowledged. First, the current study focused primarily on transcriptomic datasets, including bulk and single-cell RNA-sequencing data. Although the similarity construction methods implemented in PySimi are general and are not restricted to transcriptomic data, applying the framework to other omics modalities, such as proteomics, metabolomics, and multi-omics datasets, presents additional challenges. These data often differ in feature dimensionality, sparsity, measurement noise, and data heterogeneity, which may influence similarity construction and hyperparameter selection. Therefore, further evaluation and optimization for these data types will be an important direction for future work. Second, the present implementation focuses on spectral clustering and related visualization methods. Future work may explore the application of PySimi-generated similarity matrices to other graph-based learning and clustering approaches. Finally, automated similarity measure recommendation and parameter optimization strategies may further improve the usability and analytical performance.

In conclusion, we present PySimi, an open-source Python framework for flexible similarity matrix construction and spectral clustering. By integrating diverse similarity construction methods within a unified platform, PySimi enables systematic comparison and evaluation of similarity measures across omics datasets. The framework provides both programmatic and interactive interfaces and serves as a practical tool for clustering analysis, method evaluation, and exploratory omics research.

## Data Availability

The original contributions presented in the study are publicly available. This data can be found at https://github.com/Tianyi-Shi-Tsukuba/PySimi/tree/main/datasets.
