# Supplementary material for: PySimi: a unified framework for similarity measure evaluation in spectral clustering with applications to omics data
Source: Front Genet. 2026 Aug 19;17:1913487. doi: 10.3389/fgene.2026.1913487 (PMC13506145; doi:10.3389/fgene.2026.1913487)
Supplement: Supplementary file 1 [file DataSheet1.docx]

**Supplementary Materials for**

**PySimi: A Unified Framework for Similarity Measure Evaluation in Spectral Clustering with Applications to Omics Data**

Supplementary Materials includes:

Figures S1, Table S1.

**Figure S1.** Parameter sensitivity analysis of neighborhood size on the Inhouse single-cell RNA-seq dataset. The four panels show clustering performance measured by NMI, ARI, ACC, and Purity for different similarity construction methods.

**Table S1.** Runtime (seconds) comparison of different similarity construction methods

| Method/Dataset | Inhouse | PBMC10k | CBMC8k |
| --- | --- | --- | --- |
| Pearson | 0.06 | 1.46 | 1.63 |
| Spearman | 0.08 | 1.71 | 1.87 |
| RBF | 0.11 | 4.04 | 4.36 |
| Cosine | 0.04 | 1.32 | 1.63 |
| Polynomial | 0.04 | 1.29 | 1.59 |
| Sigmoid | 0.04 | 1.58 | 1.83 |
| Self_Tuning | 0.12 | 5.46 | 5.91 |
| iSNN | 1.67 | 61.74 | 64.55 |
| Density_Adaptive | 7.62 | 406.91 | 503.96 |
| kNN | 0.13 | 2.18 | 2.46 |
| Kernel_Adaptive | 0.28 | 10.88 | 13.01 |
| Adaptive | 0.19 | 8.07 | 9.81 |
| nSNN | 1.08 | 172.38 | 222.64 |
| cSNN | 1.08 | 169.33 | 221.93 |
